# Impact of multidimensional assessment on anti-fracture treatment decisions in patients with fragility hip fractures within a Fracture Liaison Service

**DOI:** 10.1186/s12877-025-06009-1

**Published:** 2025-05-14

**Authors:** Chiara Ceolin, Stefania Sella, Cristina Simonato, Ester Bukli, Giulia Bano, Valentina Camozzi, Anna Bertocco, Marco Onofrio Torres, Alberta Cecchinato, Martin Diogo, Mor Peleg Falb, Francesca Guidolin, Maria Grazia Rodà, Michele Cannito, Antonio Berizzi, Andrea Venturin, Vito Cianci, Elisa Pala, Mariachiara Cerchiaro, Deris Gianni Boemo, Maria Vittoria Nesoti, Gaetano Paride Arcidiacono, Paolo Simioni, Pietro Ruggieri, Giuseppe Sergi, Sandro Giannini, Marina De Rui, on behalf of the Hip-POS working group, Chiara Ceolin, Stefania Sella, Cristina Simonato, Ester Bukli, Giulia Bano, Valentina Camozzi, Anna Bertocco, Marco Onofrio Torres, Alberta Cecchinato, Martin Diogo, Mor Peleg Falb, Francesca Guidolin, Maria Grazia Rodà, Michele Cannito, Antonio Berizzi, Andrea Venturin, Vito Cianci, Elisa Pala, Mariachiara Cerchiaro, Deris Gianni Boemo, Maria Vittoria Nesoti, Gaetano Paride Arcidiacono, Paolo Simioni, Pietro Ruggieri, Giuseppe Sergi, Sandro Giannini, Marina De Rui, Carlotta Andaloro, Sara Bertolino, Davide Cannavò, Giacomo Contini, Martina Dall’ Agnol, Mario Degan, Marta Dianin, Michela Ferrarese, Claudia Finamoni, Mario Rosario Lo Storto, Elena Marigo, Stefano Masiero, Caterina Mian, Alessandra Pizziol, Giovanna Romanato, Paola Romano, Cristina Russo, Sandro Savino, Giulia Termini, Michele Tessarin, Franz Villanova, Federica Vilona, Hillary Veronese, Francesca Zanchetta, Chiara Ziliotto

**Affiliations:** 1 Geriatrics Division, Department of Medicine, University of Padua, Padua, Italy; 2 Department of Neurobiology, Care Sciences and Society, Karolinska Institutet and Stockholm University, Aging Research Center, Stockholm, Sweden; 3 Clinica Medica 1, Department of Medicine, University of Padua, Via Giustiniani 2, Padua, 35128 Italy; 4 Endocrinology Unit, Department of Medicine, Azienda Ospedale-Università Padova, Padua, Italy; 5 Department of Orthopedics and Orthopedic Oncology, D.I.S.C.O.G. Department, University of Padova, Padua, Italy; 6 Orthopedics and Traumatology Unit, Azienda Ospedale-Università Padova, Padua, Italy; 7 Physical Medicine and Rehabilitation Unit, Azienda Ospedale-Università Padova, Padua, Italy; 8 Emergency Department, Azienda Ospedale-Università Padova, Padua, Italy; 9 Department of Directional Hospital Management, Azienda Ospedale-Università Padova, Padua, Italy

**Keywords:** Multidimensional prognostic index, Comprehensive geriatric assessment, Fragility hip fractures, Fracture liaison service

## Abstract

**Background:**

Osteoporosis is a chronic condition characterized by increased fracture risk. Fragility fractures, especially hip fractures, represent a significant health and economic burden due to population aging. Despite the efficacy of approved treatments in lowering fracture recurrence, post-fracture treatment rates remain suboptimal. To address these issues, various post-fracture care programs, including Fracture Liaison Services (FLS), have been implemented worldwide. While FLS models effectively reduce refracture risk and maintain cost-effectiveness, it is unclear if these benefits apply equally to all patients, especially those with higher comorbidities and reduced functional capacity, who may face worse prognoses. This study aimed to identify the primary factors influencing anti-fracture therapy decisions in older patients with fragility fractures, using a multidimensional geriatric assessment approach integrated into our FLS program.

**Methods:**

A retrospective analysis was conducted on patients aged 65 and above with hip fractures admitted to Azienda Ospedale-Università Padova. Patients were categorized based on anti-fracture treatment (bisphosphonates, Denosumab, anabolic agents) or calcium/vitamin D supplements only. Clinical data, including the Multidimensional Prognostic Index (MPI) and its components, were collected. Statistical comparisons between treated and untreated groups were made, and a CHAID decision tree was used to explore decision-influencing factors.

**Results:**

The study included 493 patients (average age 84.7 years, 71.8% female). Patients receiving anti-fracture treatment were notably younger, with only 11.2% classified as MPI class 3 (severe prognosis) compared to 60.8% of untreated patients (*p* < 0.001). Among treated patients (*n* = 427), 75.3% received bisphosphonates, 7.3% Denosumab, and 2.2% anabolic agents. The CHAID decision tree highlighted MPI class as the primary determinant of treatment, with functional autonomy (Instrumental Activity of Daily Living or IADL) and cognitive status as subsequent factors, leading to an overall prediction accuracy of 70%.

**Conclusion:**

The integration of the MPI into multidisciplinary taking care of old patients with hip fractures may provide a structured approach for individualizing treatment decisions, considering aspects such as prognosis, functional autonomy, and cognitive status. Further studies are needed to validate the long-term outcomes of this approach.

## Introduction

Osteoporosis is a chronic disease characterized by low bone density, altered bone microarchitecture, and thus increased risk of fragility fractures (FF) [1]. The incidence of FF in European countries is expected to increase in the coming decades due to the progressive aging of the population. Particularly, in Italy hip fractures among women are projected to increase from 82,060 to 98,539, resulting in a 27% rise in associated economic costs [2]. Hip fractures are indeed the most severe type of FF because they lead to significant disability, increased hospitalization expenses, and elevated mortality rates [3]. Moreover, even ten years after a first hip fracture, the increased risk of a second hip fracture persists [4], and this occurrence may further increase mortality [5].

It is well established that approved treatments for osteoporosis are effective in reducing the incidence of subsequent fractures at various sites, with a favorable risk–benefit profile [6]. But despite these premises, the rate of anti-fracture treatment initiation after a hip fracture is still inadequate [7]. To address these issues, various post-fracture care (PFC) programs, including Fracture Liaison Services (FLS), have been implemented worldwide [8]. In 2023, we established an interdisciplinary FLS model called Hip-POS (Hip-Padua OsteoSarcopenia) at the Azienda Ospedale-Università Padova in Italy, as previously described [9]. In addition to the evaluation of traditional fracture risk factors, a key advantage of our model is the integration of a multidimensional assessment, which provides a more comprehensive characterization of patients’ comorbidities and functional autonomy across multiple domains, particularly in older adults. While many FLS models have demonstrated effectiveness in reducing refracture risk [10] and maintaining a positive cost-effectiveness balance [11], it remains unclear whether these benefits extend uniformly to all patients, especially those with higher comorbidity and poorer functional ability who may have a worse prognosis. This uncertainty highlights the need for a more nuanced approach to patient care and treatment decisions within the FLS framework.

In this context, the aim of our study is to analyze the key factors influencing the decision to administer anti-fracture therapy in older patients, with a particular focus on the components of the multidimensional geriatric assessment. By examining these factors, we aim to gain a deeper understanding of how the comprehensive evaluation offered by our Hip-POS model could be involved in the treatment decision process.

## Materials and methods

### Study population

We conducted a retrospective study on patients aged 65 years and older, admitted with hip fractures at the Azienda Ospedale-Università Padova (Italy) within our Hip-POS FLS program. The complete organization of Hip-POS was previously described [9]. Briefly, we included only patients with fragility hip fractures assessed from March 2023 to March 2024, excluding those with traumatic or pathological fractures (i.e., primary or secondary bone tumors, Paget’s bone disease).

### Clinical data collection

From all computerized medical records, we assessed during hospital admission:

- *Demographic and anthropometric characteristics*.
- *Risk factors for skeletal fragility* (e.g., previous fragility fractures, family history of fractures, smoking habit, glucocorticoid use) and calculation of the FRAX score [12]. Additionally, we collected data on previous anti-fracture treatment use.
- *Laboratory Tests*: These tests were conducted at the Laboratory Medicine Unit of the Azienda Ospedale-Università Padova, utilizing methods monitored for quality performance in accordance with the ISO 15189 standard. Lithium-heparin plasma samples were collected for calcium and phosphate measurements using a colorimetric method, and for creatinine via an enzymatic assay (calibrated to the reference procedure). Albumin was measured using an immunoturbidimetric method on the Cobas 8000 (Roche Diagnostics, Mannheim, Germany). Serum 25-OH-vitamin D and parathyroid hormone (PTH, third generation assay, reference range 6.5–36.8 ng/L) levels were measured using automated immunochemiluminescent methods (Liaison XL, DiaSorin, Saluggia, Italy).
- *Multidimensional Prognostic Index* (MPI): The MPI is a prognostic index for one-year mortality, calculated using information from the following scales (referred to the immediate period before the fracture): the Cumulative Illness Rating Scale (CIRS) for comorbidities [13], the Activities of Daily Living (ADL) [14] and Instrumental Activities of Daily Living (IADL) [15] for functional autonomy, the Mini Nutritional Assessment (MNA) [16] for nutritional status, the Short Portable Mental Status Questionnaire (SPMSQ) [17] for cognitive performance, and the Exton Smith scale (ESS) [18] for pressure sore risk. Additionally, data on the patient’s medication regimen and cohabitation status were collected. The MPI score categorizes into three risk classes: class 1 (mild risk), class 2 (moderate risk), and class 3 (severe risk). MPI was performed by a geriatrician.
- Anti-fracture treatment and/or calcium/vitamin D prescription: the therapy decision was clinically based on osteoporosis treatment guidelines and Italian prescribing regulations, as described elsewhere [9], considering factors such as previous fractures, hip fracture despite established anti-fracture treatment, or estimated 10-year fracture risk. Moreover, the patient’s condition and comorbidities were taken into account, as well as the patient’s preferences.

### Statistical analysis

Categorical variables are presented as counts and percentages, while continuous quantitative variables are expressed as mean ± standard deviation or median (interquartile range). The normal distribution of continuous variables was assessed using the Shapiro–Wilk test. The sample was divided into two groups based on the prescription of anti-fracture therapy, following osteoporosis guidelines [19] and Italian prescribing regulations [20]. Patients were categorized as either “treated” (those prescribed bisphosphonates, Denosumab, or anabolic drugs such as teriparatide and romosozumab after the FLS evaluation) or “untreated” (those not undergoing any anti-fracture therapy, only recommended calcium and/or vitamin D supplements). To compare variables between groups, quantitative variables were analyzed using the Mann–Whitney and Kruskal–Wallis tests, while categorical variables were assessed using the Chi-square test. A decision tree was built using a chi-squared automatic interaction detection (CHAID) algorithm to identify factors of geriatric multidimensional evaluation influencing treatment choices [21]. Variables with a *p* value < 0.10 in multivariate logistic regression were included in the CHAID analysis. The decision tree, a non-parametric procedure, required no assumptions about the underlying data. We set the maximum number of splits to four, the minimum number of cases in the parent node to 50, and the minimum number of cases in the child node to 20 to preserve statistical power. Node splitting was considered significant with a *p* value < 0.05 using Bonferroni’s correction. As the proportion of missing data for each variable was insignificant, multiple imputations were not used. The final model was evaluated by calculating the misclassification risk estimate and overall accuracy percentage. A tenfold cross-validation was conducted to confirm the misclassification risk for the sample. Misclassification risk refers to the incorrect classification of a patient; this risk is estimated by applying the tree to an excluded subsample. Among untreated patients, a cluster analysis was performed using the *k*-means method to identify homogeneous groups. Optimal *k* values were determined using the inertia criterion and the elbow method. Variables used included individual items from the MPI. The analysis revealed two main clusters, validated by calculating the silhouette score, which indicated good separation between clusters. All analyses were performed using the Statistical Package for Social Science (SPSS) 29.0 software (Armonk, NY: IBM Corp) with the significance level set at *p* < 0.05.

## Results

The sample characteristics are detailed in Table 1. We included 493 patients, with an average age of 84.7 years (± 7.4), and 71.8% of the patients being female. The most common comorbidities observed in the entire population were arterial hypertension (30.0%), diabetes mellitus (17.8%), ischemic heart disease (11.6%), heart failure (11.8%), chronic obstructive pulmonary disease (COPD) (8.9%), and gastrointestinal disorders (10.1%). The median number of medications taken by patients was 5.00 (IQR: 3.00–7.00), highlighting a considerable prevalence of polypharmacy. Regarding cohabitation status, most patients lived with their family (63.0%), while 32.6% lived alone and a smaller proportion (4.4%) resided in nursing homes. Stratification by MPI classified 38.9% of patients in class 1 (low risk), 43.2% in class 2 (moderate risk), and 17.8% in class 3 (high risk), indicating a significant proportion of patients with moderate to severe frailty. Regarding fracture risk, 108 patients (21.9%) had previously experienced a hip or vertebral fracture, while the 10-year probability of major osteoporotic fractures, calculated with the FRAX score, was 22.00 (14.00;31.50). Despite high fracture risk, before the index hip fracture, only 188 patients (38.1%) were taking calcium and/or vitamin D supplements, while 41 patients (8.3%) were receiving or had previously received any anti-fracture treatment.

**Table 1:** Characteristics of the sample based on prescription of anti-fracture treatment

*Variable*	*All (n* = *493)*	*Treated (n* = *427)*	*Not treated (n* = *66)*	*p-value*
*Age [years]*	84.7 ± 7.4	84.0 ± 7.4	89.3 ± 6.0	< *0.001*
*Sex, female [%]*	354 (71.8%)	304 (71.2%)	50 (75.8%)	0.55
*Cohabitative status [%]*				*0.02*
*Alone*	141 (32.6%)	128 (34%)	13 (23.6%)	
*With Family*	272 (63.0%)	236 (62.6%)	36 (65.5%)	
*Nursing homes*	19 (4.4%)	13 (3.4%)	6 (10.9%)	
*Active smokers [%]*	35 (7.1%)	33 (7.7%)	2 (3.0%)	0.14
*Comprehensive Geriatric Assessment*				
*SPMSQ*	2.00 (0.00;6.00)	2.00 (0.00;5.00)	8.00 (4.00;10.00)	< *0.001*
*ESS*	16.00 (14.00;19.00)	17.00 (15.00;19.00)	12.00 (10.00;15.00)	< *0.001*
*ADL*	5.00 (3.00;6.00)	5.00 (4.00;6.00)	1.00 (0.00;3.00)	< *0.001*
*IADL*	4.00 (0.00;7.00)	4.00 (1.00;7.00)	0.00 (0.00;1.00)	< *0.001*
*CIRS*	3.00 (2.00;5.00)	3.00 (2.00;5.00)	4.00 (3.00;6.00)	0.08
*MNA*	21.00 (17.00;24.50)	22.50 (17.63;25.00)	16.00 (10.00;18.25)	< *0.001*
*MPI classes [%]*				< *0.001*
*1*	192 (38.9%)	187 (43.8%)	5 (7.6%)	
*2*	213 (43.2%)	192 (45%)	21 (31.8%)	
*3*	88 (17.8%)	48 (11.2%)	40 (60.8%)	
*Total n.drugs*	5.00 (3.00;7.00)	5.00 (3.00;7.00)	4.00 (3.00;7.00)	< *0.001*
*Main comorbidities*				
*Hypertension*	345 (30%)	129 (30.2%)	47 (71.2%)	0.88
*Heart failure*	58 (11.8%)	43 (10.1%)	15 (22.7%)	*0.006*
*Ischemic heart disease*	57 (11.6%)	45 (10.5%)	12 (18.2%)	0.09
*COPD*	44 (8.9%)	37 (8.7%)	7 (10.6%)	0.64
*Diabetes*	88 (17.8%)	79 (18.5%)	9 (13.6%)	0.39
*Rheumatic disease*	25 (5.1%)	18 (4.2%)	7 (10.6%)	0.06
*GERD/malabsorbitive disorders*	50 (10.1%)	41 (9.6%)	9 (13.6%)	0.38

Numbers are presented as mean ± standard deviation, median (interquartile range) or number (percentage), as appropriate

*Abbreviations*: *SPMSQ* Short Portable Mental Status Questionnaire, *ESS* Exton Smith Scale, *ADL* Activities of Daily Living, *IADL* Instrumental Activities of Daily Living, *CIRS* Cumulative Illness Rating Scale, *MNA* Mini Nutritional Assessment, *MPI* Multidimensional Prognostic Index, *COPD* Chronic Obstructive Pulmonary Disease

Following the assessment conducted within our FLS program, 427 patients (86.6%) initiated anti-fracture treatment either during hospitalization or in the following weeks. We therefore categorized the patients into those who initiated and those who did not initiate anti-fracture treatment (Table 1). Those in the former group were notably younger and lived more frequently alone. Additionally, they performed better across almost all multidimensional geriatric assessment tests. Regarding the MPI, only 11.2% of the treated patients were classified as MPI class 3 (indicating a severe prognosis within one year), compared to 60.8% of the untreated patients (*p* < 0.001).

No significant differences were found between treated and untreated patients regarding osteoporosis risk factors or the prescription of anti-fracture therapy prior to the index fracture (Table 2). Regarding laboratory tests performed during hospitalization, untreated patients had lower serum levels of vitamin D [31.0 (17.0;57.0) vs. 48.0 (22.5;72.5), *p* < 0.001].

**Table 2:** Comparative analysis of treated and untreated patient cohorts, considering anamnesis of osteoporosis risk factors and calcium-phosphate metabolism

*Variable*	*Treated (n* = *427)*	*Not treated (n* = *66)*	*p-value*
*Osteoporosis risk factors [%]*			
*Family history of osteoporosis*	77 (18%)	7 (10.6%)	0.16
*Early menopause*	34 (11.2%)	2 (4.0%)	0.14
*Previous major fractures (hip/vertebral)*	95 (22.2%)	13 (19.7%)	0.72
*Glucocorticoid therapy*	10 (2.3%)	2 (3.0%)	0.67
*Previous osteoporosis therapy*			
*Vitamin D/calcium supplementation*	170 (39.8%)	18 (27.3%)	0.06
*Bisphosphonates*	36 (8.4%)	2 (3.0%)	0.40
*Denosumab or Teriparatide*	3 (0.7%)	0	0.60
*Calcium-phosphate metabolism*			
*Calcium [mg/dL]*	8.7 (8.3;9.0)	8.5 (8.2;8.8)	0.09
*Phosphate [mg/dL]*	2.9 ± 0.8	3.0 ± 0.84	0.33
*PTH [ng/L]*	35.8 (26.4;49.7)	40.3 (27.8;53.7)	0.08
*Vitamin D [nmol/L]*	48.0 (22.5;72.5)	31.0 (17.0;57.0)	*0.01*
*Albumin [g/L]*	28.0 (26.0;31.0)	27.5 (25.0;30.0)	0.11
*Creatinine [mg/dL]*	0.78 (0.62;0.97)	0.83 (0.65;1.37)	*0.01*

Numbers are presented as mean ± standard deviation, median (interquartile range) or number (percentage), as appropriate

*Abbreviations*: *MPI* Multidimensional Prognostic Index, *PTH* Parathyroid hormone

**Fig. 1:**
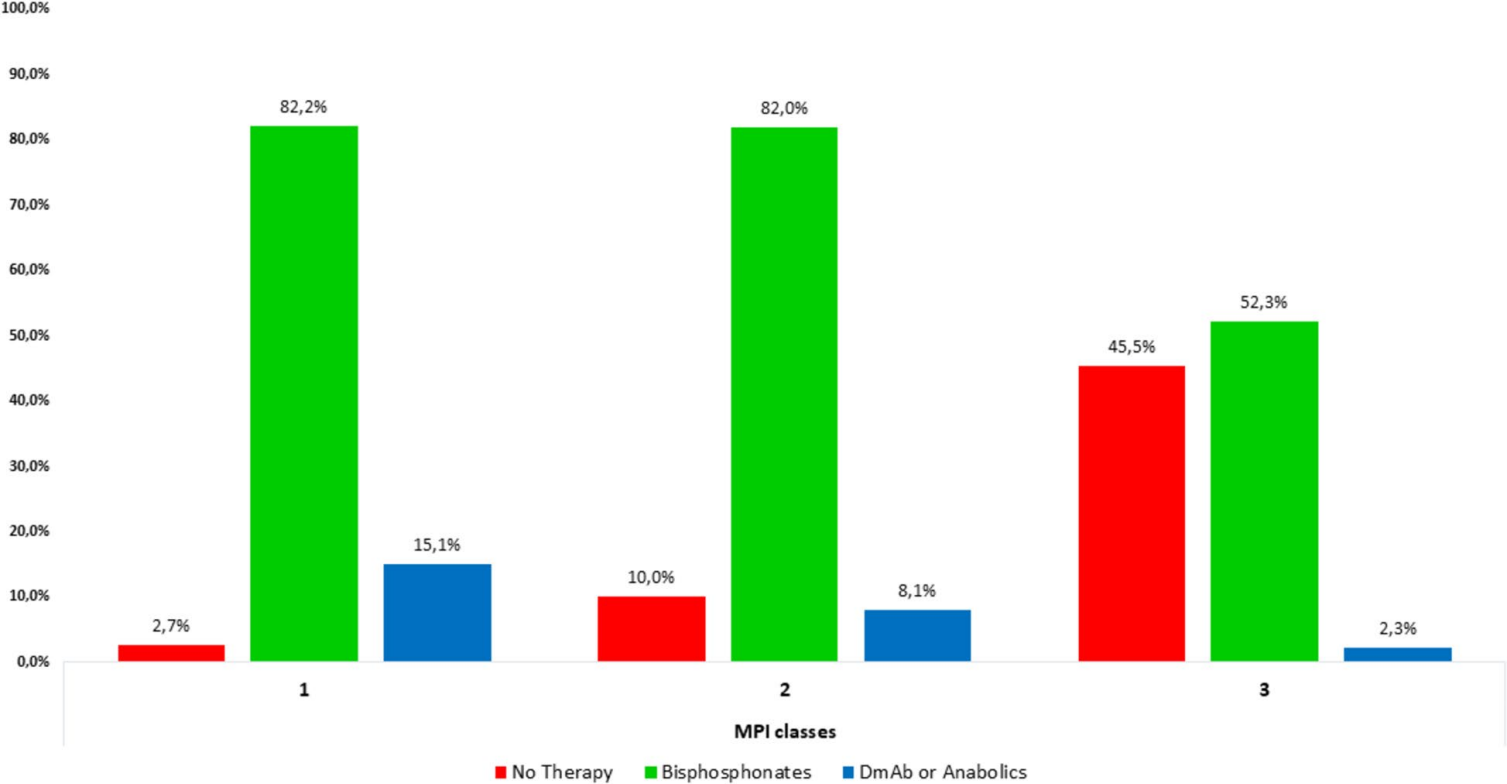
Treatment Distribution by MPI Class. MPI: Multidimensional Prognostic Index

Among the patients who received anti-fracture treatment (*n* = 427), 371 were treated with bisphosphonates (94.3% of whom received intravenous zoledronic acid), while denosumab was prescribed to 36 patients and anabolic agents to 11 patients. The remaining 9 received combination therapy. Figure 1 illustrates the distribution of anti-fracture therapy across different MPI classes. Among individuals in MPI class 1, 82.2% were treated with bisphosphonates, 15.1% received denosumab or anabolic therapies, and 2.7% did not receive any therapy. In contrast, 45.5% of patients in MPI class 3 received no treatment, while 52.3% were prescribed bisphosphonates and 2.3% received denosumab or anabolic therapies.

**Fig. 2:**
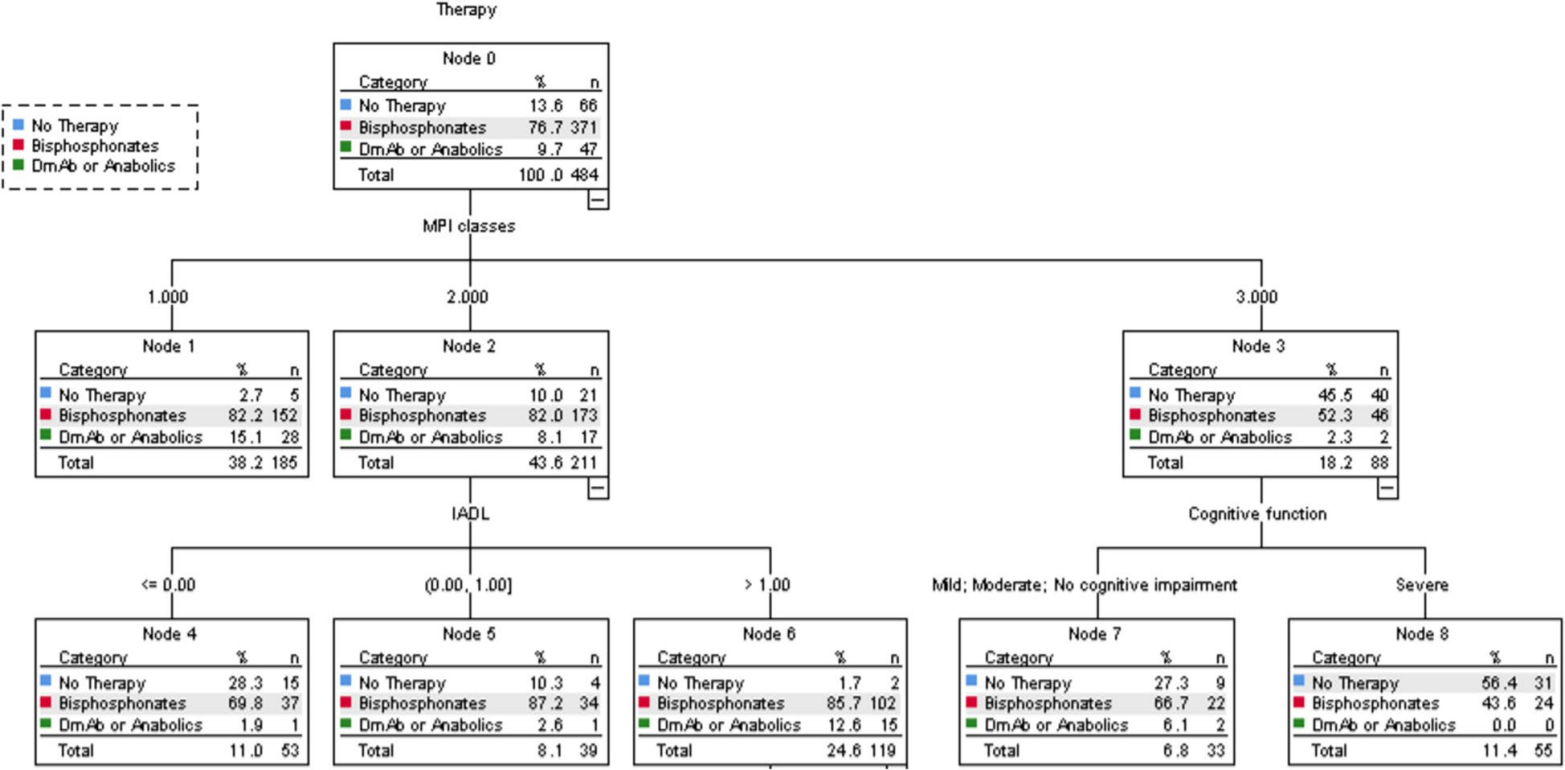
CHAID Decision Tree depicting the multifaceted decision pathway for optimizing patient therapy selection, incorporating considerations of treatment types including Bisphosphonates, Denosumab, and other anabolic agents. CHAID: Chi-square automatic interaction detection

**Fig. 3:**
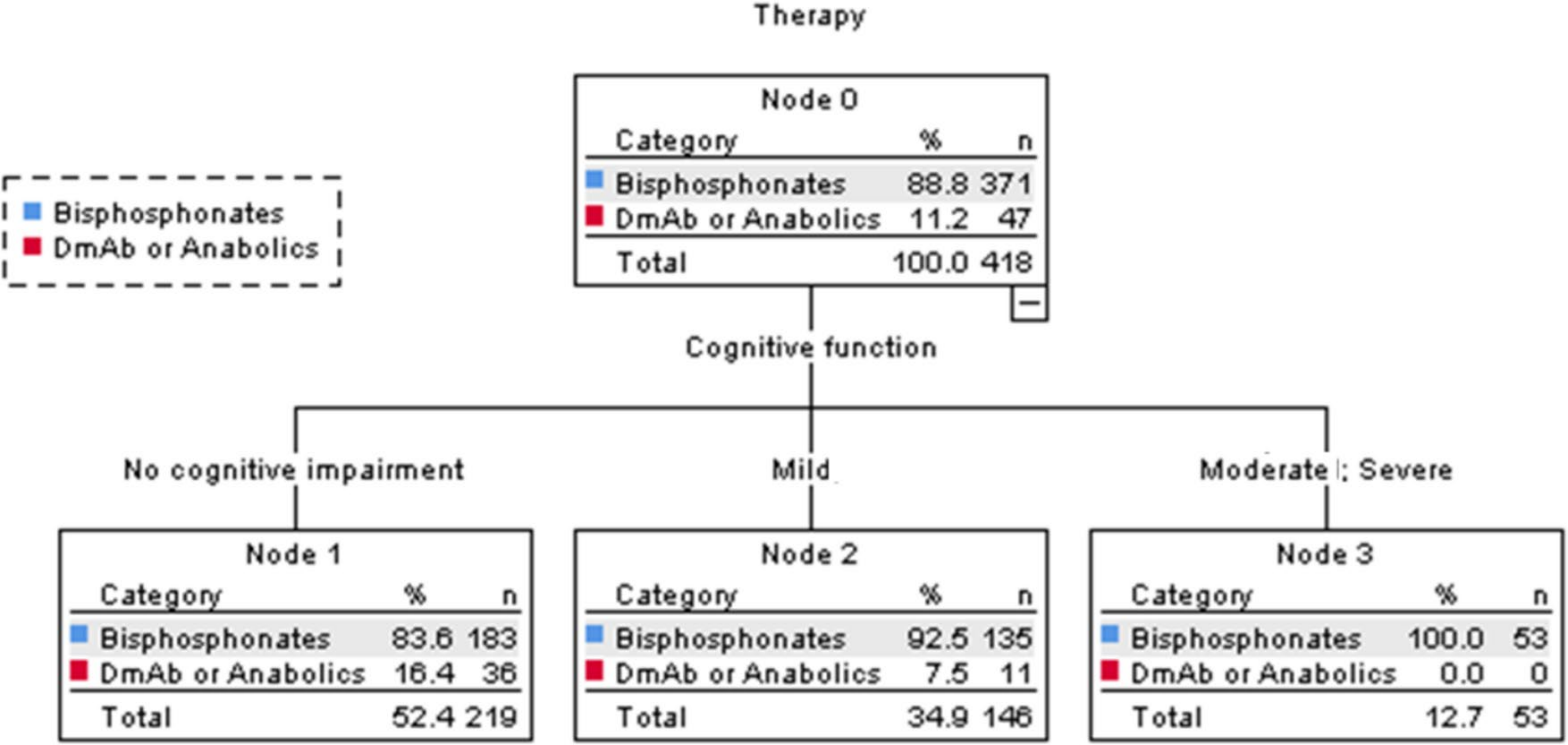
CHAID Decision Tree illustrating the role of cognitive function in selecting anti-fracture therapy, including Bisphosphonates, Denosumab, and Anabolic agents. CHAID: Chi-square automatic interaction detection

The CHAID decision tree (Fig. 2) illustrates the factors that retrospectively influenced the clinical decision to initiate treatment, based on multidimensional geriatric evaluation items. The primary decision was based on MPI values. Among patients in MPI class 1, 82.2% were treated with bisphosphonates, while 15.1% received other treatments, including denosumab or anabolic agents. In contrast, only 2.3% of patients in MPI class 3 received denosumab or anabolic medications, with 45.5% receiving only vitamin D and calcium supplementation. For those with MPI class 2, the next criterion was functional ability, assessed by IADL scores. Among patients with IADL > 1, 12.6% received denosumab or anabolic treatment compared to only 1.9% of those with IADL = 0. For patients with MPI class 3, cognitive function was the next factor considered. Approximately 27.3% of patients with intact or mild cognitive impairment did not receive treatment, a rate that rose to 56.4% among those with severe cognitive impairment. Additionally, 6.1% of patients with intact or mild cognitive decline received denosumab or anabolic agents, while none of those with severe cognitive impairment received this type of treatment. The risk estimate for the decision tree was 0.300, the standard error 0.019, which means that this classification tree analysis was able to predict the decision to treat patients or not with an accuracy of approximately 70%. The decision tree had a sensitivity of 94% and a specificity of 30%. Among the treated patients, cognitive function was the only factor from the geriatric assessment that influenced the choice of therapy type (Fig. 3). Specifically, 16.4% of patients with normal cognitive function received denosumab or anabolic agents, whereas only 7.5% of those with mild or severe cognitive impairment were prescribed these medications.

## Discussion

In this study, we retrospectively analyzed the primary factors influencing the decision to administer anti-fracture therapy, with a particular focus on the multidimensional geriatric assessment. Our analysis identified three key elements: patient prognosis; functional autonomy, reflecting the capacity to independently perform daily activities; and cognitive status, evaluated in terms of the ability to manage and adhere to therapy independently. This approach not only allows for anti-fracture treatment personalization based on the true capabilities and needs of the old patient but also facilitates the anticipation of potential barriers to treatment effectiveness, such as adherence challenges or risks associated with frailty.

Regarding the decision to initiate an anti-fracture treatment in the “oldest old”, this has been a matter of debate for years, largely because most clinical trials on anti-fracture drugs have involved younger subjects, and evidence of anti-osteoporotic efficacy in the oldest old has mainly come from subgroup analyses [22]. Despite the high fracture risk, older patients are often under-treated for skeletal fragility, mainly due to safety concerns and the perception that anti-fracture efficacy requires long-term treatment [22]. In treated older patients, bisphosphonates represent the most commonly used class [23]. A meta-analysis of 23,287 women over the age of 65 confirmed that bisphosphonates are safe for older adults, and the reported adverse effects, particularly with zoledronic acid, are generally mild or moderate and should not discourage their prescription [24]. Furthermore, a large meta-analysis observed that the time-to-benefit of bisphosphonate therapy was 12.4 months to prevent one nonvertebral fracture per 100 women with postmenopausal osteoporosis, suggesting that bisphosphonate therapy is most likely to benefit individuals with a life expectancy greater than 12.4 months [25]. Given these premises, the decision to initiate anti-fracture treatment should not be based solely on age but should also consider functional autonomy, cognitive status, and, most importantly, patient prognosis, elements typical of a multidimensional geriatric assessment.

A crucial aspect that emerged from our study is the central role of the MPI in the decision-making process regarding anti-fracture therapy in older adults. The MPI, a widely adopted prognostic tool, assesses patient frailty in hospitalized individuals through a multidimensional model [26–28]. This index not only takes into account biological and physiopathological aspects but also integrates clinical outcomes and manifestations such as functional deficits, reduced mobility, cognitive decline, loss of independence in daily activities, and the presence of multiple chronic conditions [29]. Although multidimensional assessments have been integrated into orthogeriatric care models, helping to identify frail individuals at higher risk of fractures [30], there are currently no specific studies that directly link the MPI to time-to-benefit for anti-osteoporosis or fracture treatments. For instance, higher MPI values are associated with an increased risk of both vertebral and non-vertebral fractures, as well as a higher incidence of hospitalization-related complications (e.g., delirium) [31]. In this context, our study represents a pioneering effort, but further research is needed to validate the long-term outcomes of this approach and to better understand how the MPI may influence the timing and effectiveness of anti-fracture therapies. In our study, we observed that patients with MPI scores of 1 and 2, indicative of a more favorable prognosis, received anti-fracture therapy significantly more often, with bisphosphonate prescription rates around 80%. In contrast, only 45.5% of patients with severe MPI, indicating a less favorable prognosis, received less intensive treatments, such as supplementation with calcium and vitamin D. This disparity highlights the emphasis clinicians place on prognostic factors, focusing more intensive interventions on patients with a longer life expectancy. The approach centered on patients with a better prognosis serves a dual purpose: on one hand, it aims to maximize the long-term benefits of anti-fracture therapy for those most likely to benefit; on the other hand, it seeks to minimize the risk of adverse effects in patients with unfavorable prognoses, where the risks of treatment may outweigh the benefits [32, 33]. This strategy aligns with the current trend of incorporating life expectancy and prognosis as key factors in evaluating the benefits and risks of tests and treatments [34]. However, despite the availability of various prognostic tools, evidence supporting their routine use to improve clinical outcomes has been limited thus far. Our study aims to partially address this gap by demonstrating that the MPI is a potentially effective prognostic tool for guiding therapeutic decisions in the geriatric population.

After assessing the prognostic aspect, particularly in cases of intermediate risk, another crucial element that should guide therapeutic decisions is the patient’s functional status. Indeed, pre-fracture functional status is a significant predictor of post-fracture outcomes in older adults with hip fractures: patients with better pre-fracture function tend to experience more favorable outcomes and have a higher likelihood of regaining mobility [35]. Our decision tree revealed that patients with a pre-fracture IADL score greater than 1 were treated in nearly all cases. This is because patients with higher IADL scores typically exhibit better baseline autonomy, greater potential for recovery, and a more favorable prognosis, along with a longer life expectancy [36]. In contrast, patients with lower IADL scores may be more frail and therefore more vulnerable to the side effects of medications, making treatment potentially riskier [37]. Preferring treatment for patients with greater functional autonomy not only reduces the risk of complications but also increases the likelihood of proper adherence to the prescribed therapy, thereby optimizing treatment effectiveness [38].

Finally, the third aspect to consider is cognitive functioning, which represents the second step in the decision to administer anti-osteoporotic therapy in patients with severe MPI. Patients with significant cognitive impairment rarely received treatments with Denosumab or anabolic drugs, while approximately 56% received supplementation with vitamin D and calcium. In the absence of a caregiver, forgetfulness, misinterpretation of instructions, and difficulties in adhering to complex dosing schedules contribute to high rates of non-adherence to therapy among cognitively impaired patients [39]. Moreover, the progressive decline in cognitive function impairs the ability to plan, organize, and execute the tasks necessary for proper medication management, increasing the risk of unintentional non-adherence and medication errors [40]. In line with this, the decision tree indicates that the only factor influencing the prescription of Denosumab or anabolic drugs was the cognitive domain, with these medications being more frequently prescribed to cognitively intact individuals. Our results can likely be explained by the method of drug administration: an annual i.v. injection is undoubtedly more convenient for patients with cognitive decline, who might face significant difficulties managing medications on their own without assistance. Consistent with findings from other researchers, this facilitates the administration and prescription of anti-fracture therapy for individuals with cognitive decline, where the risk of fractures should not be underestimated [41].

Among the limitations of our study is its retrospective design, which may introduce biases or inaccuracies. Another limitation concerns the fact that the choice of anti-fracture therapy may also be influenced by local prescription regulations. Specifically, prescription rules in Italy are likely more restrictive than those suggested by International Guidelines regarding the use of anabolic medications, even in patients at high risk of fracture. We also acknowledge that a proportion of patients did not receive anti-fracture treatment, which clearly represents an area for potential improvement in our program. Furthermore, we could not establish the actual effectiveness of our approach. Additional research will be necessary to monitor patient survival during follow-up and, consequently, to evaluate the efficacy of our therapeutic decision-making strategy.

In conclusion, our study highlights three key factors that should guide the decision to initiate anti-fracture therapy: prognostic, functional, and cognitive aspects. This emphasizes the critical role of a multidimensional approach in orthogeriatric care. Further data, particularly from larger populations and extended follow-up periods, are needed to validate these findings and determine whether comprehensive assessments are truly essential for optimizing clinical decisions and improving patient outcomes.

## Data Availability

The datasets analysed during the current study are available from the corresponding author on reasonable request.

## References

[1] Kanis JA. Diagnosis of osteoporosis. Osteoporos Int a J Establ as result Coop between Eur Found Osteoporos Natl Osteoporos Found USA. 1997;7(Suppl 3):S108–16. 10.1007/BF03194355 9536315

[2] Borgström F, Karlsson L, Ortsäter G, Norton N, Halbout P, Cooper C, et al. Fragility fractures in Europe: burden, management and opportunities. Arch Osteoporos. 2020;15(1):59. 32306163 10.1007/s11657-020-0706-y PMC7166207

[3] Veronese N, Maggi S. Epidemiology and social costs of hip fracture. Injury. 2018;49(8):1458–60. 29699731 10.1016/j.injury.2018.04.015

[4] Sobolev B, Sheehan KJ, Kuramoto L, Guy P. Risk of second hip fracture persists for years after initial trauma. Bone. 2015;75:72–6. 25681701 10.1016/j.bone.2015.02.003

[5] Sobolev B, Sheehan KJ, Kuramoto L, Guy P. Excess mortality associated with second hip fracture. Osteoporos Int a J Establ as result Coop between Eur Found Osteoporos Natl Osteoporos Found USA. 2015;26(7):1903–10. 10.1007/s00198-015-3104-3 25910745

[6] Händel MN, Cardoso I, von Bülow C, Rohde JF, Ussing A, Nielsen SM, et al. Fracture risk reduction and safety by osteoporosis treatment compared with placebo or active comparator in postmenopausal women: systematic review, network meta-analysis, and meta-regression analysis of randomised clinical trials. BMJ. 2023;381:e068033. 37130601 10.1136/bmj-2021-068033 PMC10152340

[7] Kim SC, Kim M-S, Sanfélix-Gimeno G, Song HJ, Liu J, Hurtado I, et al. Use of osteoporosis medications after hospitalization for hip fracture: a cross-national study. Am J Med. 2015;128(5):519-26.e1. 25660252 10.1016/j.amjmed.2015.01.014 PMC4414898

[8] Åkesson KE, Ganda K, Deignan C, Oates MK, Volpert A, Brooks K, et al. Post-fracture care programs for prevention of subsequent fragility fractures: a literature assessment of current trends. Osteoporos Int a J Establ as result Coop between Eur Found Osteoporos Natl Osteoporos Found USA. 2022;33(8):1659–76. 10.1007/s00198-022-06358-2 PMC8943355 35325260

[9] Arcidiacono GP, Ceolin C, Sella S, et al. Taking care of inpatients with fragility hip fractures: the hip-padua osteosarcopenia (Hip-POS) fracture liaison service model. J Endocrinol Invest 2025;48:99–108. 10.1007/s40618-024-02425-z. 10.1007/s40618-024-02425-z PMC11729073 38971949

[10] Li N, Hiligsmann M, Boonen A, van Oostwaard MM, de Bot RTAL, Wyers CE, et al. The impact of fracture liaison services on subsequent fractures and mortality: a systematic literature review and meta-analysis. Osteoporos Int a J Establ as result Coop between Eur Found Osteoporos Natl Osteoporos Found USA. 2021;32(8):1517–30. 10.1007/s00198-021-05911-9 PMC8376729 33829285

[11] Wu C-H, Kao I-J, Hung W-C, Lin S-C, Liu H-C, Hsieh M-H, et al. Economic impact and cost-effectiveness of fracture liaison services: a systematic review of the literature. Osteoporos Int a J Establ as result Coop between Eur Found Osteoporos Natl Osteoporos Found USA. 2018;29(6):1227–42. 10.1007/s00198-018-4411-2 29460102

[12] Kanis JA, Harvey NC, McCloskey E, Bruyère O, Veronese N, Lorentzon M, et al. Algorithm for the management of patients at low, high and very high risk of osteoporotic fractures. Osteoporos Int a J Establ as result Coop between Eur Found Osteoporos Natl Osteoporos Found USA. 2020;31(1):1–12. 10.1007/s00198-019-05176-3 PMC7018677 31720707

[13] Linn BS, Linn MW, Gurel L. Cumulative illness rating scale. J Am Geriatr Soc. 1968;16(5):622–6. 10.1111/j.1532-5415.1968.tb02103.x. 10.1111/j.1532-5415.1968.tb02103.x 5646906

[14] Katz S. Assessing self-maintenance: Activities of daily living, mobility, and instrumental activities of daily living. J Am Geriatr Soc. 1983;31(12):721–7. 6418786 10.1111/j.1532-5415.1983.tb03391.x

[15] Graf C. The lawton instrumental activities of daily living scale. Am J Nurs [Internet]. 2008 Apr [cited 2023 Jan 13];108(4):52–62. Available from: https://pubmed.ncbi.nlm.nih.gov/18367931/. 10.1097/01.NAJ.0000314810.46029.74 18367931

[16] Vellas B, Guigoz Y, Garry PJ, Nourhashemi F, Bennahum D, Lauque S, et al. The mini nutritional assessment (MNA) and its use in grading the nutritional state of elderly patients. Nutrition [Internet]. 1999 Feb [cited 2023 Jan 13];15(2):116–22. Available from: https://pubmed.ncbi.nlm.nih.gov/9990575/. 10.1016/s0899-9007(98)00171-3 9990575

[17] Pfeiffer E. A Short Portable Mental Status Questionnaire for the Assessment of Organic Brain Deficit in Elderly Patients. J Am Geriatr Soc [Internet]. 1975 [cited 2023 Jan 13];23(10):433–41. Available from: https://pubmed.ncbi.nlm.nih.gov/1159263/. 10.1111/j.1532-5415.1975.tb00927.x 1159263

[18] Pilotto A, Ferrucci L, Franceschi M, D’Ambrosio LP, Scarcelli C, Cascavilla L, et al. Development and validation of a multidimensional prognostic index for one-year mortality from comprehensive geriatric assessment in hospitalized older patients. Rejuvenation Res [Internet]. 2008 Feb 1 [cited 2023 Jan 13];11(1):151–61. Available from: https://pubmed.ncbi.nlm.nih.gov/18173367/. 10.1089/rej.2007.0569 PMC2668166 18173367

[19] Castañeda S, Navarro Ceballos C, Usón Jaeger J, de Miguel Benadiba C, Gómez Martín E, Martínez Díaz-Guerra G, Alvarez-Galovich L. Management of Vertebral Fragility Fracture in Older People: Recommendations from a Spanish Consensus of Experts. Geriatr. 2024;9(2):24. 10.3390/geriatrics9020024. 10.3390/geriatrics9020024 PMC10961758 38525741

[20] Nuti R, Brandi ML, Checchia G, Di Munno O, Dominguez L, Falaschi P, et al. Guidelines for the management of osteoporosis and fragility fractures. Intern Emerg Med. 2019;14(1):85–102. 29948835 10.1007/s11739-018-1874-2 PMC6329834

[21] Song Y-Y, Lu Y. Decision tree methods: applications for classification and prediction. Shanghai Arch psychiatry. 2015;27(2):130–5. 26120265 10.11919/j.issn.1002-0829.215044 PMC4466856

[22] Rizzoli R, Branco J, Brandi M-L, Boonen S, Bruyère O, Cacoub P, et al. Management of osteoporosis of the oldest old. Osteoporos Int a J Establ as result Coop between Eur Found Osteoporos Natl Osteoporos Found USA. 2014;25(11):2507–29. 10.1007/s00198-014-2755-9 25023900

[23] Delli Poggi C, Fusaro M, Mereu MC, Brandi ML, Cianferotti L. Cardiovascular Safety and Effectiveness of Bisphosphonates: From Intervention Trials to Real-Life Data. Nutrients. 2022;14(12):2369. 10.3390/nu14122369. 10.3390/nu14122369 PMC9227734 35745099

[24] Fan Q, Wang J. The Efficacy and Safety of Bisphosphonates for Osteoporosis in Women Older Than 65 Years: A Meta-Analysis. Curr Pharm Des. 2020;26(32):4022–30. 10.2174/1381612826666200423092602. 10.2174/1381612826666200423092602 32324507

[25] Deardorff WJ, Cenzer I, Nguyen B, Lee SJ. Time to Benefit of Bisphosphonate Therapy for the Prevention of Fractures Among Postmenopausal Women With Osteoporosis: A Meta-analysis of Randomized Clinical Trials. JAMA Intern Med. 2022;182(1):33–41. 34807231 10.1001/jamainternmed.2021.6745 PMC8609461

[26] Bureau ML, Liuu E, Christiaens L, Pilotto A, Mergy J, Bellarbre F, et al. Using a multidimensional prognostic index (MPI) based on comprehensive geriatric assessment (CGA) to predict mortality in elderly undergoing transcatheter aortic valve implantation. Int J Cardiol [Internet]. 2017;236:381–6. Available from: https://pubmed.ncbi.nlm.nih.gov/28238508/. Cited 2023 Jan 13. 10.1016/j.ijcard.2017.02.048 28238508

[27] Hansen TK, Shahla S, Damsgaard EM, Bossen SRL, Bruun JM, Gregersen M. Mortality and readmission risk can be predicted by the record-based Multidimensional Prognostic Index: a cohort study of medical inpatients older than 75 years. Eur Geriatr Med. 2021;12(2):253–61. 33570735 10.1007/s41999-021-00453-z

[28] Mattace-Raso F, Pilotto A. The challenge of the multifaceted prognosis in the older people and the Multidimensional Prognostic Index. Eur Geriatr Med. 2021;12(2):223–6. 10.1007/s41999-021-00457-9. 10.1007/s41999-021-00457-9 PMC7900797 33620704

[29] Pilotto A, Custodero C, Maggi S, Polidori MC, Veronese N, Ferrucci L. A multidimensional approach to frailty in older people [Internet]. Vol. 60, Ageing Research Reviews. Ageing Res Rev; 2020 [cited 2023 Jan 13]. Available from: https://pubmed.ncbi.nlm.nih.gov/32171786/. 10.1016/j.arr.2020.101047 PMC7461697 32171786

[30] Veronese N, Smith L, Zigoura E, Barbagallo M, Dominguez LJ, Barone A, et al. Multidimensional prognostic index and the risk of fractures: an 8-year longitudinal cohort study in the Osteoarthritis Initiative. Arch Osteoporos. 2021;17(1):5. 34905117 10.1007/s11657-021-01015-3 PMC8669664

[31] Musacchio C, Custodero C, Razzano M, Raiteri R, Delrio A, Torriglia D, et al. Association between multidimensional prognostic index (MPI) and pre-operative delirium in older patients with hip fracture. Sci Rep. 2022;12(1):16920. 36209284 10.1038/s41598-022-20734-2 PMC9547845

[32] Pilotto A, Azzini M, Cella A, Cenderello G, Castagna A, Pilotto A, et al. The multidimensional prognostic index (MPI) for the prognostic stratification of older inpatients with COVID-19: A multicenter prospective observational cohort study. Arch Gerontol Geriatr. 2021;95:104415. 33882420 10.1016/j.archger.2021.104415 PMC8020604

[33] Salis F, Cossu E, Mandas A. The multidimensional prognostic index (MPI) predicts long-term mortality in old type 2 diabetes mellitus patients: a 13-year follow-up study. J Endocrinol Invest. 2024;47(1):191–200. Available from: 10.1007/s40618-023-02135-y. 10.1007/s40618-023-02135-y PMC10776747 37332086

[34] Veronese N, Fazzari A, Armata M, Parisi A, Parrinello A, Petralia V, et al. Clinical prognostic factors for older people: a systematic review and meta-analysis. Ageing Res Rev. 2024;98:102345. 38777131 10.1016/j.arr.2024.102345

[35] Ceolin C, Bano G, Biz C, Dianin M, Bedogni M, Guarnaccia A, et al. Functional autonomy and 12-month mortality in older adults with proximal femoral fractures in an orthogeriatric setting: risk factors and gender differences. Aging Clin Exp Res. 2023;35(5):1063–71. 36892795 10.1007/s40520-023-02378-y

[36] Prestmo A, Saltvedt I, Helbostad JL, Taraldsen K, Thingstad P, Lydersen S, et al. Who benefits from orthogeriatric treatment? Results from the Trondheim hip-fracture trial. BMC Geriatr. 2016;16(1):1–10. Available from: 10.1186/s12877-016-0218-1. 10.1186/s12877-016-0218-1 PMC4761133 26895846

[37] Manias E, Kabir MZ, Maier AB. Inappropriate medications and physical function: a systematic review. Ther Adv drug Saf. 2021;12:20420986211030372. 10.1177/20420986211030371 PMC8287273 34349978

[38] Fratino L, Polesel J, Giunta EF, Maruzzo M, Buti S, Hassan MA, et al. Instrumental activities of daily living in older patients with metastatic prostate cancer: results from the meet-URO network ADHERE prospective study. Sci Rep. 2024;14(1):1–8. Available from: 10.1038/s41598-024-53581-4. 10.1038/s41598-024-53581-4 PMC10902368 38418470

[39] Elliott RA, Goeman D, Beanland C, Koch S. Ability of older people with dementia or cognitive impairment to manage medicine regimens: a narrative review. Curr Clin Pharmacol. 2015;10(3):213–21. 26265487 10.2174/1574884710666150812141525 PMC5396255

[40] He X, Wang X, Wang B, Zhu A. The Association Between Mild Cognitive Impairment and Medication Non-adherence Among Elderly Patients With Chronic Diseases. Cureus. 2023;15(10):e47756. 37899893 10.7759/cureus.47756 PMC10602820

[41] Prieto-Alhambra D, Judge A, Arden NK, Cooper C, Lyles KW, Javaid MK. Fracture prevention in patients with cognitive impairment presenting with a hip fracture: secondary analysis of data from the HORIZON Recurrent Fracture Trial. Osteoporos Int a J Establ as result Coop between Eur Found Osteoporos Natl Osteoporos Found USA. 2014;25(1):77–83. 10.1007/s00198-013-2420-8 PMC3867338 23812596

